# Is There a Relationship between Adverse Pregnancy Outcomes and Future Development of Atherosclerosis?

**DOI:** 10.3390/biomedicines11092430

**Published:** 2023-08-31

**Authors:** Anastasia V. Poznyak, Victoria A. Khotina, Elena B. Zhigmitova, Vasily N. Sukhorukov, Anton Y. Postnov, Alexander N. Orekhov

**Affiliations:** 1 Institute for Atherosclerosis Research, Osennyaya 4-1-207, 121609 Moscow, Russia; 2 Laboratory of Cellular and Molecular Pathology of Cardiovascular System, Federal State Budgetary Scientific Institution «Petrovsky National Research Centre of Surgery» (FSBSI “Petrovsky NRCS”), Abrikosovsky per., 2, 119991 Moscow, Russia; nafany905@gmail.com (V.A.K.); lenae7777@gmail.com (E.B.Z.); vnsukhorukov@gmail.com (V.N.S.); anton-5@mail.ru (A.Y.P.)

**Keywords:** atherosclerosis, pregnancy, gestation, cardiovascular risk factors

## Abstract

Cardiovascular disease is one of the main death causes globally. Effective cardiovascular risk management requires a thorough understanding of the mechanisms underlying the disorder. Establishing early markers of the disease allows a timely intervention and prevention of further atherosclerosis development. Multiple studies confirm the correlation between pregnancy disorders and cardiovascular disease in the postpartum period. Moreover, over 30% of women experience adverse pregnancy outcomes. Thus, the examination of the links between these conditions and atherosclerotic cardiovascular disease may help to identify gender-specific risk factors. In this review, we will explore the association between several adverse pregnancy outcome conditions and atherosclerosis. The current analysis is based on the data from several recent studies on the mechanisms behind gestational diabetes, hypertensive disorders of pregnancy, miscarriages, and stillbirths and their implications for the female cardiovascular system.

## 1. Pregnancy Implications for Cardiovascular Disease

In the United States, cardiovascular disease (CVD) is the primary reason behind female fatalities, and recognizing gender-specific determinants could improve cardiovascular risk evaluation and management. Being pregnant is a distinct exposure only women undergo as about 85% of females deliver offspring, and almost a third might encounter an adverse pregnancy outcome (APO) [1]. Conditions related to APOs, such as hypertension-related pregnancy disorders (HPD), pregnancy diabetes, premature delivery, reduced birth weight, and atherosclerotic cardiovascular disease (ASCVD) in older age, have been researched only lately [2].

ASCVD risk factors in later life, as well as a higher incidence of diagnosed ASCVD, have been demonstrated to correlate with the prevalence of the above-mentioned conditions. This is why the American Heart Association advises that an assessment of APOs be included in the evaluation of ASCVD risk [3]. Furthermore, the 2018 guidelines for cholesterol management recommend considering pregnancy-related disorders as risk enhancers while evaluating the use of statins for ASCVD treatment in females. The 2016 CVD prevention guidelines from the European Society of Cardiology assert that it is uncertain if APOs are risk determinants themselves or whether they promote other conditions that aggravate atherosclerosis in female patients [4,5].

## 2. Cardiovascular Abnormalities in Pregnancy

**Figure 1:**
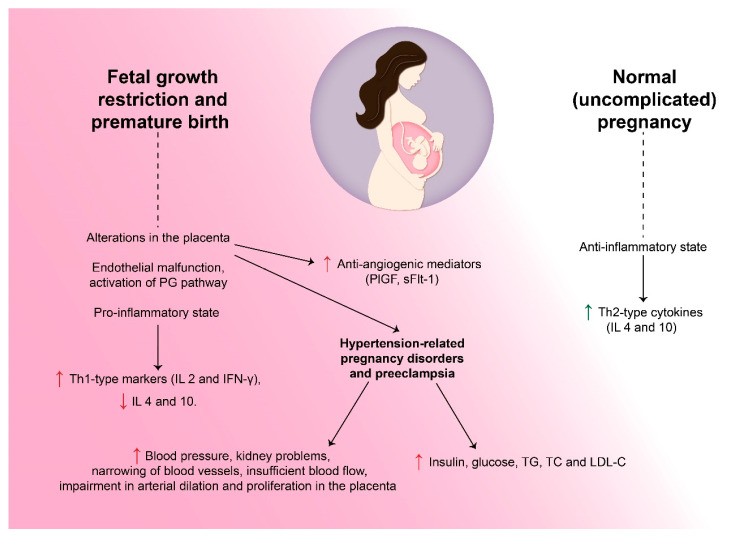
Vascular and metabolic changes during both normal and complicated pregnancies. In a normal pregnancy, the immune system undergoes an anti-inflammatory shift to support the developing fetus. However, complications in pregnancy can lead to a pro-inflammatory state, endothelial dysfunction, and alterations in the placenta. These changes can result in increased blood pressure followed by many pathological conditions associated with the development of pre-eclampsia and hypertension-related pregnancy disorders. Abbreviations: IL—interleukin; LDL-C—low-density lipoprotein cholesterol; PG—prostaglandin; PIGF—placental growth factor; sFlt-1—soluble receptor of PIGF; TC—total cholesterol; TG—triglycerides.

The female cardiovascular system is challenged by the vascular and metabolic changes during pregnancy that signify a potential cardiovascular risk and may lead to gestational complications. In Figure 1, we proposed a slight comparison of normal and complicated pregnancy in the scope of cardiovascular risks. These disorders are viewed as a failed cardiovascular stress test, necessitating early prevention and a proactive risk modification approach [6]. This review looks into the physiological changes in pregnancy that mimic atherosclerosis and offers some guidelines for avoiding complications in the future. A meta-study summarizing the data of 22 studies examined the biochemical transformations that occur as a result of HPD, revealing that increased levels of insulin, glucose, triglycerides (TG), total cholesterol (TC), and low-density lipoprotein cholesterol (LDL-C) remained after pregnancy. The average follow-up period was 16.9–23 years, showing that metabolic disorders persist after HDP [7].

Fetal growth restriction (FGR) and premature birth have been associated with endothelial malfunction, activation of prostaglandin pathway, and the presence of inflammatory cytokines. During an uncomplicated pregnancy, the immune system changes to an anti-inflammatory state to support the developing fetus. Studies indicate that the presence of inflammatory markers may result in premature birth [8]. In a normal pregnancy, Th2-type cytokines are dominant (such as interleukin (IL4) and interleukin (IL10)). However, pregnancies with underdeveloped fetuses exhibited an increase in Th1 markers, including interleukin 2 and IFN-γ, and a decrease in IL 4 and 10. This pro-inflammatory state and endothelial dysfunction in pregnancies with premature birth and impaired growth of the fetus resemble ASCVD [9].

### 2.1. Specific Placental Factors

The development of pre-eclampsia and HDP can be initiated by changes to the placenta, inflammation, endothelial dysfunction, and genetic factors. Pre-eclampsia and eclampsia are brought on by alterations in the placenta and result in high blood pressure, kidney problems, narrowing of blood vessels throughout the body, and insufficient blood flow. Additionally, with further damage to blood vessels, platelets become active, the endothelium constricts, and blood coagulators are released [10]. Recent studies have discovered anti-angiogenic mediators created by the placenta that alter the mother’s vascular function and are likely due to inadequate blood flow and abnormal implantation of the placenta. Recently, two angiogenic factors have been discovered: the placental growth factor (PlGF) and its soluble receptor (sFlt-1). SFlt-1 receptor binding to vascular and placental growth factors reduces growth factor levels and causes endothelial constriction. sEng is another soluble protein that influences the blood vessels’ ability to dilate in response to nitric oxide. At the moment, scientists aim to find out the factors promoting the excess release of these proteins in the placenta. Their release can be set off by oxidation, hypoxemia, and immunologic factors [11].

Various studies show that women with preterm PE have lower levels of PIGF in the first trimester, compared to women with normal gestation. Moreover, an association was observed between the PIGF concentration in serum and the severity of PE. The value of PlGF and sFlt-1/PlGF as exclusion tests for the diagnosis and short-term prediction of PE has been well established in singleton pregnancies, surpassing traditional clinical parameters in determining time to delivery and predicting adverse perinatal outcomes [12].

Recent investigations revealed that microRNAs take part in the physiological regulation, as well as pathological development of placenta in patients with PE. Expression of microRNA varies in normal placentas and placentas of women with PE. In clinic, the changes (upregulation and downregulation) in different miRNAs were observed in placentas with PE [13]. For example, aberrant expression of those miRNAs in human trophoblast cells gives rise to their arrested proliferation and inadequate invasion, which further lead to the failure in sufficient remodeling of maternal spiral arteries and possibly consequent deficiency in angiogenesis. The miRNAs with significantly up-regulated expression in PE placenta, such as miR-17, miR-155, miR-431, and others, were found to negatively regulate trophoblast cell proliferation, migration, invasion, and apoptosis [14].

Some studies have shown that miRNAs expression is linked to GDM occurrence, and aberrant expression of miRNAs in human placenta leads to the pathogenesis of GDM, such as miR-296, an expression of which is improperly down-regulated in placenta with GDM in clinic and affects human placental development through the alterations in migration and invasion of trophoblast cells by regulating HIF3A [15]. 

Although the use of biomarkers in labor planning in women with PE has not yet been adequately investigated and is not recommended, there is evidence that knowledge of PlGF levels can reduce serious maternal morbidity. Further research is needed to establish the benefits of planned early birth for perinatal outcomes using PlGF-based testing as more than just supplementing other clinical information in decision making [16]. Evidence is emerging of the potential benefits of angiogenic biomarkers for the diagnosis and prognosis of other complications, including fetal growth retardation and stillbirth, and this is a research priority given the association of these complications with pre-eclampsia [17].

### 2.2. Constriction of Blood Vessels

Constriction of blood vessels is a crucial factor in pre-eclampsia-related issues, such as increased blood pressure, impaired kidney function, and excessive activation of the sympathetic nervous system. Unlike uncomplicated pregnancy, blood flow and filtration in the kidneys are reduced during pre-eclampsia. An impairment in arterial dilation and proliferation can be observed in the placenta [18]. The constriction of blood vessels in the placenta and reduced blood flow cause long-term oxygen deprivation in the fetus and placenta, which can result in hypertensive disorders of pregnancy, premature birth, stillbirth, and fetal growth restriction (FGR) [19].

## 3. Pregnancy Complications

**Figure 2:**
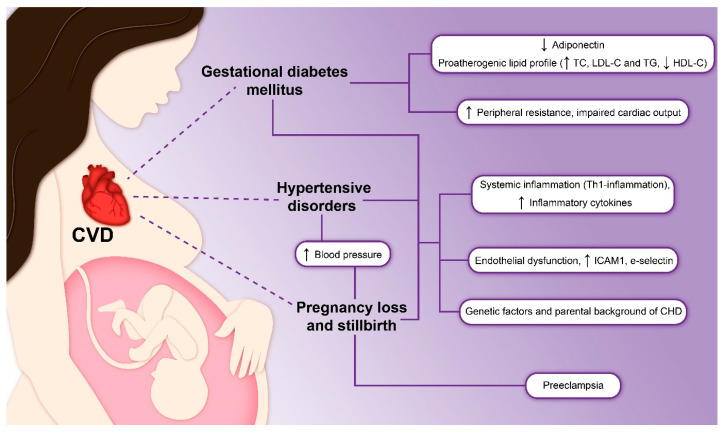
Maternal cardiovascular complications in pregnancy: a comprehensive scheme.

In this section, we reviewed the most widespread pregnancy complication that are associated with CVD risks. In Figure 2, we provided the scheme of our current understanding of cause-effect relationship.

### 3.1. Gestational Diabetes

Gestational diabetes mellitus (GDM) is a diabetic condition identified during the second or third trimester of pregnancy, with or without remission following delivery. GDM occurrence has been reported to range from 1% to 28% and is on the rise, particularly in developed nations. GDM can contribute to adverse outcomes both for the mother and the fetus and result in later onset of diabetes mellitus 2 (DM2) after the delivery [20]. Furthermore, females with GDM also have an increased risk of developing CVD later in life. Many lifestyles risk factors as well as non-modifiable risk factors contribute to the association between GDM and related CVD, including high blood sugar, glucose intolerance, unfavorable lipid profiles, advanced age, and higher levels of C-reactive protein (CRP). Specifically, the onset of diabetes carries higher risk of CVD in later life [21].

However, the precise mechanisms that connect GDM and CVD are yet to be identified. Metabolic dysfunctions, such as unfavorable lipid profile and vascular impairment, are commonly seen in female patients with a history of previous GDM (pGDM) several years later. These women exhibit high levels of inflammatory cytokines, low adiponectin, high peripheral resistance, and impaired cardiac output [22]. In comparison with healthy women of the same age, pGDM patients also have elevated TC, LDL-C, and TG as well as lower HDL-C, indicating a proatherogenic profile. In most cases, obesity, sedentary lifestyle, and genetic factors likely account for the initiation of CVD in these women [23].

Carotid artery intima-media thickness (CIMT) is an indicator of atherosclerosis that has a significant correlation with the risk of cardiovascular events, especially in females. Additionally, it anticipates the emergence of cardiovascular disease from gestational diabetes mellitus [24].

Multiple studies have been conducted recently on females who have experienced pGDM, revealing that they have elevated levels of endothelial dysfunction indexes and CIMT compared to control groups, even without any obvious metabolic irregularities. Earlier research has also demonstrated a correlation between various genetic variations and cardiovascular as well as metabolic risk factors in females, emphasizing the necessity of taking these factors into account along with commonly evaluated markers like lipid levels during pregnancy for their involvement in the progression of GDM-related CVD [25].

Numerous standard indicators and biochemical indexes are currently at our disposal to measure the possibility of diabetes in pGDM females’ post-pregnancy. Despite the low prevalence of cardiovascular events in young patients, identifying potential risk of coronary microvascular dysfunction at an early stage can offer a crucial chance for timely management and prevention [26]. Enhancing follow-ups after delivery is necessary to reduce not only the prevalence of diabetes but also the related CVD, as many researchers have underscored the connection between well-established CVD risk factors and gestational diabetes. Evaluating CIMT and arterial stiffness can be crucial in determining the likelihood of heart disease and strokes, in addition to traditional markers [27].

Bo et al. evaluated CIMT in 82 females with pGDM and 113 without, after six and a half years post-delivery [28]. Their study disclosed that pGDM females, regardless of their BMI and the existence of metabolic disorders, exhibited significantly higher inter-cellular adhesion molecule 1, e-selectin, and intima-media thickness (IMT) indexes than the control group. IMT was found to have a notable correlation with pGDM following adjustments for blood sugar, body mass index, waist circumference, and blood pressure. Volpe et al. examined CIMT in 28 women with pGDM and 24 without, two years after pregnancy, discovering that young females who had a history of gestational diabetes presented early markers of inflammation in the vessels, but within upper normal levels [29]. The primary metabolic syndrome (MS) constituents varied between the pGDM women and controls, including TG levels, waist circumference, blood pressure, and blood glucose in fasting, with all the rates being remarkably higher in the pGDM group.

A 1.4-fold increased incidence of CVD associated with GDM was observed by Kaul et al. [30]. Retnakaran and Shah later affirmed comparable outcomes, highlighting that GDM females are exposed to an increased risk of adverse cardiovascular consequences, even without diabetes [31].

Elevated TG and reduced high-density lipoprotein cholesterol (HDL-C) levels are commonly found in DM2. It is not unexpected that some trials found these characteristics in pGDM females. While the cardiovascular (CV) risk implications of high TG levels and low HDL-C are still debated, the cardiovascular relevance of LDL-C and apolipoprotein B (apoB) is well known [32]. Earlier research showed that GDM females on the third trimester displayed notably higher TC and LDL-C compared to the controls, and an association was established between the lipid profile and specific gene polymorphisms in Apolipoprotein A5 (APOA5) and LDLR; TG levels were also elevated in GDM females, although the difference was not statistically relevant. Remarkably, a more recent study reported a correlation between TG levels on the third trimester and carotid intima media thickness (*p* = 0.014) [33].

The noteworthy outcomes of this study are consistent with the findings by Di Cianni et al., who observed that cholesterol levels, both total and LDL, as well as blood glucose and blood pressure were all remarkably elevated in the GDM group, indicating that these women had a profile resembling metabolic syndrome (MS) [34]. These specific modifications in the lipoprotein parameters may promote gestational endothelial dysfunction. The lipid parameters seen in GDM females are comparable to that of individuals with insulin resistance. 

#### 3.1.1. Gestational Diabetes and Associated Cardiovascular Risks

Gongora and Wenger demonstrated that pGDM females display a proatherogenic lipidogram three months after delivery, with higher CIMT than in the control group, and elevated MS risk [35].

It is acknowledged that pregnancy imposes a strain on the body, as high blood sugar levels appear to have a substantial influence on the cardiovascular system during this brief period; we might suggest that lipid changes that occur during pregnancy with GDM are a source of harm that contributes to CVD risk in later life. However, the underlying mechanisms of this correlation require further examination. GDM females demonstrate a predominance of small dense LDL particles; moreover, they are more prone to LDL oxidation [36].

These findings underscore the importance of effectively and continuously monitoring females with pGDM to mitigate both metabolic and cardiovascular risks. Unfortunately, the data indicate insufficient postpartum glucose monitoring. Moreover, postpartum screening of pGDM patients remains inadequate, necessitating the development of an efficient and easy-to-implement tool that can include both panel genes and metabolic profile analysis to identify patients at high risk for DM and CVD that require more in-depth medical supervision. Earlier research showed that elevated cardiovascular risk in pGDM females in the postpartum period results from an interaction of genetic predisposition and diet [37].

#### 3.1.2. Genetic Predispositions

Moreover, it is vital to establish the characteristics of the examined gene variations. Remarkably, an earlier Italian trial revealed that APOA5-1131T>C could influence the risk of premature MI with a 1.44 odds ratio (CI: 1.23–1.69) per C allele. The apolipoprotein A-V gene is situated near the APOAI, APOCIII, and APOA-IV gene cluster on human 11q23. ApoAV is determined by the APOA5 [38]. ApoAV is produced in the liver and is transported by chylomicrons, very low-density lipoprotein (VLDL), and HDL. Standard variation in the APO genes can affect the lipid profile. 

A meta-analysis newly demonstrated a connection between APOA5 rs662799 C allele and TG concentrations in subjects of all ethnic groups. This might mean that circulating TG acts as a mediator between APOA5 high risk and atherosclerotic conditions. Various research noticed correlation of APOA5 risk with decreased HDL-C and increased triglycerides in plasma. New evidence about APOA5-1131T>C, which still requires further research, proposes that APOA5 gene might also affect blood atherogenicity directly [39]. Framingham Heart Study reported that women with C allele of the −1131T>C demonstrated almost a double increase of CVD risk. Additionally, patients with overweight and obesity demonstrated a connection between the rare allele of the −1131T>C SNP and intima-media thickness [40].

Qiao et al. noticed that DM2 patients with TC and CC genotypes showed higher triglycerides and the TG/HDL-C ratio than patients with TT genotype (*p* < 0.05). Moreover, DM2 subjects with CC genotype demonstrated higher CIMT compared to subjects with TT genotype (*p* = 0.08), though these findings are not statistically relevant [41].

A strong connection was observed between carotid IMT and APOA5 CC genotype (*p* = 0.045). Up-to-date research demonstrates that a higher cardiovascular risk is determined by a combination of various reasons including age and/or genetic factors, but the share of each factor is still to be defined. It has been found that C genotype females, even at an early age, might be more susceptible to cardiovascular disorders due to an elevated carotid IMT [42].

The low-density lipoprotein receptor (LDLR) gene can control the metabolism of cholesterol. Comprehensive multiple studies of the rs2228671, one of genetic variants at the LDL receptor locus, demonstrated its strong connection with total cholesterol and LDL-C; moreover, the T allele showed a coherent connection with a lower risk of coronary artery disease. In earlier studies, it was found that among patients with gestational diabetes, those who carried rs2228671 T allele demonstrated strong correlation with LDL-C level in the third trimester of pregnancy [43].

Additionally, a noteworthy correlation was noticed between CC APOA5/CC LDLR interplay and CIMT (*p* = 0.010). Astonishingly, females possessing the CC genetic makeup in APOA5 rs662799, without the T safeguarding allelic variant of LDLR rs2228671, exhibit an increase in CIMT. It would be captivating to further scrutinize the influential molecular process responsible for this interaction. We can speculate that the interplay effect of LDLR rs2228671 and APOA5 rs662799 entails a probable alleviation of CIMT measurements [44].

#### 3.1.3. Management of Gestational Diabetes

As articulated by Mecacci et al., gestational diabetes can be deemed a “preview of forthcoming health” where adopting a wholesome lifestyle is advisable to avert or impede diabetes and/or cardiac diseases in postnatal period [45].

Previously, it was determined that women afflicted with gestational diabetes possessed a greater body mass index compared to the control group during both the pregestational and prenatal phases. During a recent analysis, the way of life the women had at the follow-up was examined: the pGDM cohort had an average BMI in the overweight category, as well as a higher average waist measurement (86.2 ± 16.1 cm). Furthermore, no variance in adherence to the MedDiet, physical activity, and smoking habits was noted, although an opposing association between both the IPAQ and the PREDIMED scores with the CIMT measurements was observed [46]. It means that, potentially, preventive activities may be beneficial. Indeed, the sample had a 7.5 median MedDiet score (moderate adherence), meaning a feeble attachment to salubrious dietary practices; additionally, a considerable percentage reported inferior levels of physical activity. These alterable risk factors can be effortlessly addressed to facilitate early CVD prevention.

From this perspective, customized nourishment and way of life recommendations constitute a promising tactic for the prevention and handling of insulin resistance syndrome. In this respect, the primary objectives are to accurately recognize and stratify GDM women vulnerable to CVD and assess the preventive measures, as well as improve screening after childbirth [47].

Studies of extended duration are recommended to establish the potential of lifestyle change. Monitoring after gestational diabetes can be complemented with comparable quality and responsibility measures including discussions of possible risks between women and doctors as well as referral to primary healthcare [48].

This is presumably the first trial utilizing multi-sectoral inventive biomarkers to assess the risk of cardiometabolic disease in women with pregestational diabetes. On this account, women with pGDM could be incorporated into follow-up programs tailored to guarantee consistent supervision, thus delivering efficient prevention of both cardiovascular disease and type 2 diabetes (DM2) [49].

It would be of clinical value to have a risk indicator during gestation to enable the focal point of long-term follow-ups and appropriate intervention strategies focused on women at the highest risk in due time.

### 3.2. Hypertensive Disorders of Pregnancy

Chronic high blood pressure, high blood pressure during pregnancy, and combined pre-eclampsia or eclampsia are collectively known as hypertensive disorders of pregnancy (HDP). The occurrence of HDP has risen by 25 percent in the past decade and is projected to increase further due to the surge of risk factors associated with HDP development, including obesity, diabetes mellitus, and geriatric pregnancy. Consequently, there is an anticipated growth in the rates of adverse pregnancy outcomes linked with HDP, such as restricted fetal growth, prematurity, and stillbirth [50]. In the United States, HDP is a cause of 7.4 percent of maternal deaths associated with the risk of ischemic infarction, kidney failure, respiratory distress syndrome (RDS), and pulmonary edema. Women with HDP are also more vulnerable to developing coronary vascular disease at older age. Although the precise underlying pathology for the increased risk of CVD is not clear, contemporary research indicates three possible processes: (1) CVD risk induced by pregnancy, (2) a predisposition before pregnancy to an elevated CVD risk, or (3) a merging of both processes [51].

#### 3.2.1. HDPs and Cardiovascular Risk

The assessment of HDPs’ role in maternal CVD risk has gained increasing attention. Stuart et al. have recently shown that women with HDPs are at a higher CVD risk than a control group with normal blood pressure [52]. Furthermore, studies have demonstrated higher rates of subclinical ASCVD in women with HDPs in the anamnesis. Nonetheless, observational data are inadequate to definitely infer causality due to the possible influence of deficiently measured confounding and bias. The findings of another recent study contribute to the existing data by utilizing Mendelian randomization. Since Mendelian randomization has limited confounding and bias effects, these findings endorse a possible causal pathway and the increasing acknowledgement of HDPs as risk CVD factors specific for women [53].

The relationship with CVD disclosed in this research seems to be more conspicuous in the overall HDP exposure, with a tendency towards weaker effects for pregnancy hypertension and even weaker effects for pre-eclampsia and eclampsia, despite the outcomes still being statistically relevant. This can be potentially explained in many ways. Notably, the HDP events encompass already existing elevated blood pressure [54]. Therefore, there may be underlying biological processes that explain the relationship with CVD in this group of patients. Yet, comprehending this tendency in detail is challenging; the techniques utilized in different approaches differ significantly across HDP events, and hence variability in the strength of relationship may be anticipated.

The relationship between HDPs and heart failure (HF) or atrial fibrillation (AF) was not identified according to the study. On the other hand, certain observational studies have found that gestational hypertensive disorders, specifically pre-eclampsia, are related to increased rates of peripartum cardiomyopathy and HF at older ages [55]. The absence of results might be attributed to several factors. Firstly, the GWAS data incorporated all kinds of HF, and the ensuing heterogeneity in the results may have decreased the study’s power. Secondly, the genuine impact of HDPs on HF may be less significant than what was possible to detect: the authors of the study had a power of 80 percent or higher to discover an association only if there was an actual underlying relative risk increase of 11 percent for HDP events, 10 percent for pregnancy hypertension, and 5 percent for pre-eclampsia/eclampsia on HF [56]. Furthermore, the inconsistency could result from residual confounding in the study setting. This is especially accurate for pre-eclampsia, which is recognized to have extensive social, behavioral, and multifactorial drivers.

#### 3.2.2. HDPs and Atherosclerosis

Numerous investigations have tackled the topic of the correlation between HDP and asymptomatic atherosclerosis. The term “asymptomatic atherosclerosis” pertains to the inflammation of the coronary/carotid artery when it is still in an incipient stage, but ASCVD can be precisely detected and assessed by various non-invasive and invasive methods, like ultrasound, MRI, CTI, and coronary angiography [57]. According to a cohort study, having a background of pre-eclampsia raised the likelihood of coronary artery calcifications (CAC) more than thirty years after the pregnancy, even after individual controlling for conventional risk factors, though the correlation was not more substantial when adjusted for current elevated blood pressure. The authors discovered that the probability of having a higher CAC score was 3.54 (CI: 1.39–9.02) times higher in women with previous PE than in those who did not develop PE, without any modifications, and it was 2.61 (CI: 0.9–7.14) times greater after adjusting for existing hypertension [58]. On the other hand, the relationship between CAC score and the history of pre-eclampsia persisted, even after considering BMI alone (odds: 3.20; CI: 1.21–8.49). It has also been demonstrated that 33% of participants with pre-eclampsia display signs of coronary atherosclerosis on vascular CTI, in contrast to only 20% of women in the reference group CTI. This finding is noticeable as early as between ages 45 and 55, when women are typically 16 ± 6 years postpartum. Conversely, a case-control study discovered that the “average maximum CIMT was comparable between women with and without pre-eclampsia (0.831 mm vs. 0.817, *p* = 0.38). Pre-eclampsia did not prove a significant predictor of CIMT in a multiple linear regression model (*p* = 0.63), despite more ECGs indicating CHD”. The aforementioned study’s authors defined “abnormal electrocardiograms” as those that displayed at least one of the following: the presence of isolated infarct, Q waves, new left bundle branch block (LBBB), higher or lower ST than normal, or T wave inversion. The traditional common CIMT measurement does not demonstrate this, unlike measuring the individual CCA intima and media thicknesses that indicate increased vascular risk [59].

### 3.3. Pregnancy Loss and Stillbirth

There is multiple evidence, based on more than a million cases, that pregnancy loss and stillbirth were connected with higher occurrence of heart stroke, ischemic stroke, and renal hypertension. While stillbirths demonstrated higher risks compared to miscarriages, the rate increase was the same in women with four and more pregnancy losses in the anamnesis [60]. Generally, more cases of pregnancy loss in the anamnesis corresponded to an increase in occurrence of atherosclerotic complications, with a similar increase for all three of them. It was impossible to compare the corresponding statistics for stillbirths due to a limited number of those. Incidence rate ratios for ischemic stroke and renal hypertension were higher in cases of recurrent miscarriage compared to non-recurrent miscarriage, although with borderline difference. The relationship was not affected by adjustments for possible confounding factors [61].

#### 3.3.1. Miscarriages and Cardiovascular Risks

Earlier studies associated consecutive miscarriages with a higher risk of myocardial infarction (MI) and coronary heart disease (CHD) in family history. A Heidelberg cohort (including more than 10 thousand women) of a large multicenter EPIC study also noted strong correlation between miscarriages and MI. Correlation between miscarriages and cerebral infarction was not revealed. However, the study had certain limitations as there were few events and the history of miscarriages was recorded for only 40 percent of the study participants and by participants themselves [62].

A causal relationship between pregnancy losses and conditions related to ASCVD has been established in a recent study. These different issues may have similar underlying processes, probably of genetic basis, or pregnancy loss triggers a pathological mechanism leading to ASCVD which may develop in three different locations in the body without reference to any specific organ [63]. There is evidence that miscarriage is similar to autoimmune diseases as it is associated with immune mechanisms like, for example, Th1-inflammation, and various autoimmune disorders like psoriasis, chronic inflammation of the gastrointestinal tract, and rheumatoid arthritis have been linked to a higher risk of ASCVD. Immune cells, like activated T cells and macrophages, as well as signaling molecules triggering a Th1 response, are present in plaques on the endothelial walls [64]. There is evidence that recurrent miscarriages have been related to endothelial dysfunction. Hence, inflammation can potentially be a genetically based mechanism that is common to both pregnancy losses and atherosclerotic diseases.

It is uncertain whether the processes behind the links noted for miscarriage and stillbirth overlap or if distinct processes are at work. Even though early and late miscarriages have different causes, there exist factors that raise the risk of pregnancy loss during the whole pregnancy, such as fetal genetic disorders, fetal structural changes, mother’s infection and diabetes, as well as chronic illnesses [65]. It should be noted that dysfunction of endothelium caused by systemic inflammation seems to be a credible underlying mechanism shared by miscarriage (especially late and repetitive miscarriage), stillbirth, and the atherosclerotic outcomes. This vascular disorder would likely both impact placental function during gestation, causing pregnancy loss, and increase the chances of MI, ischemic stroke, and renal hypertension. In this context, a higher correlation found with stillbirth (a 74 to 169 percent increase in risk related to stillbirth versus a 13 to 20 percent increase related to miscarriage) could mean that placental issues are responsible for a bigger proportion of stillbirths than miscarriages [66]. Since the primary contributors to miscarriage in general are infections, fetal genetic irregularities, and uterine and reproductive disorders, none of which are expected to be connected to later atherosclerotic consequences in women, the overall estimations of miscarriage were more affected by miscarriages whose origins were not linked to ASCVD later in life. Still, miscarriages caused by infections and fetal genetic irregularities are likely to be occasional events, while those caused by vascular disorders are likely to repeat. In line with this assumption, we noted more robust connections for consecutive miscarriage, implying that women who experience late or consecutive miscarriages are likely to have influenced the overall estimates [67].

#### 3.3.2. Miscarriage and Later Cardiovascular Outcome

Although vascular disorders present a feasible association between pregnancy losses and the atherosclerotic consequences we analyzed, it is challenging to establish whether pre-eclampsia, a recognized hazard for subsequent CHD and ischemic stroke, is causally connected or whether miscarriage or stillbirth is autonomously related to later outcomes [68]. Pre-eclampsia is acknowledged to reoccur; hence, early pre-eclampsia or its precursors cannot be eliminated as a potential source of, for instance, MI linked with repetitive miscarriage. Nevertheless, as per definition, miscarriage happens before pre-eclampsia can be diagnosed, and therefore, this is intricate to disentangle, especially in register-based research. Correspondingly, pre-eclampsia probably contributed to some of the stillborn cases in the cohort, but due to a limited number of stillbirths classification by the assumed cause was impossible [69].

Certain processes could additionally contribute to the connections with pregnancy loss and fetal demise. Hormonal aspects and unusual maternal immune responses may reasonably connect miscarriage, but not stillbirth, to the consequences, while elevated blood pressure in a woman may provide a connection for stillbirth, but not miscarriage. Nevertheless, such hypotheses are entirely conjectural and would demand clinical information unavailable in our records to authenticate or reject [70]. 

Inherited types of MI are commonly recognized to manifest at earlier ages than non-hereditary forms. As a result, the recent discovery by Smith and coworkers, demonstrating a relationship between a parental background of CHD and consecutive pregnancy loss in daughters (a relationship that was resistant to correction for possible confounding variables like socio-economic issues and smoking), is particularly intriguing [71]. It proposes a feasible genetic association between these two disorders. Earlier studies exploring the relationships between pregnancy losses and the risk of CHD and cerebral infarction had insufficient data for examining these links in young females in particular. Analyzing three distinct atherosclerotic outcomes, the authors of a recent study discovered the most robust relationships with pregnancy losses in the youngest females. Taken with the Smith and colleagues’ discoveries, these results endorse a genetic aspect to the correlation between pregnancy loss and ASCVD. They suggest that past pregnancy losses must be taken into account when evaluating the risk of ASCVD, in younger women in particular [72].

Successive and non-successive miscarriages were contrasted since recurrent successive miscarriages may differ causally from miscarriages occurring between successful childbirths. The study reported a tendency towards stronger connection with ischemic stroke and renal hypertension for successive miscarriages compared to non-successive miscarriages. Conversely, the relationships between miscarriage and MI were alike for successive and non-successive miscarriages. Therefore, these findings do not fully endorse the concept that solely successive miscarriages are connected with heightened rates of atherosclerotic outcomes later on [73].

## 4. Conclusions

In this review, we have looked into main pregnancy complications and their correlation with the development of atherosclerosis-related conditions in older age. The findings of recent studies of adverse pregnancy outcomes were summarized. The research allowed us to conclude that gestational diabetes, hypertensive pregnancy disorders, miscarriage, and stillbirth are all associated with the onset of ASCVD later in life even after the adjustment for traditional confounding factors.

GDM and ASCVD appear to have a similar underlying genetic mechanism. Specific polymorphisms in APOA5 and LDLR result in an unfavorable lipid profile and endothelial dysfunction which promotes both diabetes and cardiovascular diseases. HPD have also been associated with CAC and CHD. However, it is still unclear whether these conditions share the same predisposing factors, or pregnancy complications negatively affect the cardiovascular system. Stillbirths and miscarriages are presumably associated with adverse cardiovascular events later in life through common genetic mechanisms that lead to systemic inflammation. Inflammatory processes have an unfavorable impact on both the pregnancy outcome and the cardiovascular system.

There is ample evidence indicating the correlation between APOs and enhanced risk for atherosclerosis in later life. However, the corresponding data are somewhat limited due to a number of reasons. Firstly, earlier studies of pregnancy complications have not been adjusted for all cardiovascular confounding factors. Some trials were not prospective, and the follow-up period was too short, while others involved patients with various ethnic backgrounds. In addition, the majority of the studies evaluated only one of the APOs and its influence on ASCVD development, although a single patient may suffer from multiple complications at the same time.

All in all, adverse pregnancy outcomes should be considered as potential gender specific markers of ASCVD that can develop in the postpartum period. Thus, more in-depth follow up practices and monitoring methods should be introduced in order to identify females at greater risk for CVD. This would allow for more timely intervention and ASCVD prevention or mitigation.

## Data Availability

Not Applicable.
